# Prenatal recruitment of participants for a birth cohort study including cord blood collection: results of a feasibility study in Bremen, Germany

**DOI:** 10.3205/000208

**Published:** 2015-04-14

**Authors:** Sinja Alexandra Ernst, Kathrin Günther, Torsten Frambach, Hajo Zeeb

**Affiliations:** 1 Leibniz-Institute for Prevention Research and Epidemiology – BIPS, Bremen, Germany; 2 St. Joseph-Stift Krankenhaus, Bremen, Germany

**Keywords:** umbilical cord blood, leukemia, children, feasibility study, birth cohort study

## Abstract

**Background: **Prospective birth cohort studies comprising follow up of children from pregnancy or birth over a long period of time, and collecting various biological samples at different times through the life-course offer a promising approach to enhance etiologic knowledge of various diseases. Especially for those where early lifetime exposures and conditions are thought to play an important role. The collection and storage of biological samples is a critical component in epidemiological studies, notably for research regarding prenatal exposures to various environmental factors as well as for DNA extraction. Our feasibility study for a birth cohort within the scope of etiology of childhood leukemia with prospective sampling of mothers and their future newborns aimed to investigate the willingness of pregnant women to participate in a birth cohort study involving collection of blood and umbilical cord blood samples. The overall aim was to develop practice-based research recommendations for a possible German birth cohort study.

**Methods: **The study was conducted in Bremen, Germany, between January 2012 and March 2013. Pregnant women were eligible for recruitment if (i) their expected date of delivery was during the study recruitment phase (September 2012–February 2013), (ii) they planned to give birth at the cooperating hospital’s obstetric unit and (iii) their knowledge of the German language was sufficient to understand study materials, details of participation and to fill out the prenatal self-administered questionnaire. Maternal blood and umbilical cord blood samples to be used for later research activities were collected and stored at a stem cell bank already collaborating with the hospital. 22 primary care gynecologists were invited to enroll pregnant women for the study and cooperation with one hospital was established. Expectant women were recruited during the last trimester of pregnancy, either during one of their prenatal care visits at their primary care gynecologist or later on in hospital by the attending obstetricians or project staff.

**Results: **Of the 22 invited primary care gynecologists requested to enroll pregnant women for the study, 8 gynecologists actually collaborated. A total of 200 eligible women were invited to participate in the study, 48 (24%) of whom agreed. 34 women were enrolled by primary care gynecologists, with one gynecologist enrolling 26 women. Twelve of 14 women recruited via hospitals were enrolled by study staff. A total of 41 women consented to the collection of umbilical cord blood and maternal blood samples, and samples could be stored for 54% of them. Reason for non-participation were the uncertainty whether or not the full study would be conducted and the fact that the participants were not willing to decide for their children whether or not genetic information (cord blood) can be stored for research purposes.

**Conclusion: **Enrolling parents in a birth cohort study that includes biosampling is a challenge, but participation can be improved through close collaboration with primary care gynecologists and maternity hospitals. Cord blood collection may impede participation, especially when maternity hospitals offer an alternative option for cord blood donation.

## Zusammenfassung

**Hintergrund: **Geburtskohorten stellen vielversprechende Forschungsansätze dar, um prospektiv unter *Life-Course*-Perspektive Ursachen und Mechanismen zahlreicher Erkrankungen zu erforschen. Die Entnahme von Bioproben, einschließlich Nabelschnurblutproben, zu verschiedenen Zeitpunkten im Lebensverlauf (insbesondere prä- und perinatal) ist von zentraler Bedeutung für eine direkte Expositionserfassung. Gleichzeitig ist die Entnahme von Bioproben als besonders kritischer Aspekt in epidemiologischen Studien zu betrachten, insbesondere für die Erforschung von pränatalen Expositionen durch umweltbezogene Faktoren und für DNA-Extraktion. Ziele dieser Machbarkeitsstudie zur Ursachenforschung für kindliche Leukämien war die Erstrekrutierung von werdenden Müttern sowie ihren zukünftigen Neugeborenen für eine Geburtskohorte, welche die Entnahme von Blut der Mutter sowie Nabelschnurblut beinhaltet. Übergreifendes Ziel war die Entwicklung von Empfehlungen für eine mögliche nationale Geburtskohorte auf Basis praktischer Erfahrungen.

**Methoden: **Die Machbarkeitsstudie wurde in Bremen im Zeitraum Januar 2012 bis März 2013 durchgeführt. Werdende Mütter wurden um Studienteilnahme gebeten, wenn (i) der geplante Geburtstermin im Erhebungszeitraum lag, (ii) die Entbindung in der kooperierenden Geburtsklinik stattfinden sollte sowie (iii) die Sprachkenntnisse ausreichend waren, um die Studieninhalte zu verstehen und den pränatalen Fragebogen auszufüllen. Blut der Mutter sowie Nabelschnurblut wurde für spätere Forschungsaktivitäten gesammelt und in einer Stammzellbank eingelagert, die bereits eine bestehende Kooperation mit der Geburtsklinik hatte. 22 niedergelassene Gynäkologinnen und Gynäkologen wurden kontaktiert und gebeten werdende Mütter in die Studie einzuschließen. Die Rekrutierung erfolgte im letzten Schwangerschaftsdrittel, entweder im Rahmen der Schwangerenvorsorge bei niedergelassenen Gynäkologinnen oder Gynäkologen oder in der Geburtsklinik über Klinikmitarbeiterinnen und -mitarbeiter oder direkt durch Projektmitarbeiterinnen.

**Ergebnisse: **8 von 22 eingeladenen niedergelassenen Gynäkologinnen und Gynäkologen konnten für die Rekrutierung gewonnen werden. Insgesamt wurden 200 werdende Mütter angesprochen und um Studienteilnahme gebeten, 48 (24%) stimmten einer Teilnahme zu. 26 der 34 werdenden Mütter, die über niedergelassene Gynäkologinnen und Gynäkologen rekrutiert wurden, wurden durch denselben Gynäkologen rekrutiert. 12 der 14 über die Geburtsklinik rekrutierten Teilnehmerinnen wurden durch Projektmitarbeiterinnen rekrutiert. 41 Frauen waren mit einer Nabelschnurblutentnahme einverstanden und für 54% konnten Proben eingelagert werden. Angegebene Gründe für die Nicht-Teilnahme waren die Unsicherheit, ob die Hauptstudie zustande kommt und dass die Teilnehmerinnen nicht für das ungeborene Kind entscheiden wollten, ob genetische Informationen (Nabelschnurblut) für Forschungszwecke eingelagert werden darf.

**Schlussfolgerung: **Die Rekrutierung werdender Mütter im Rahmen von Geburtskohortenstudien mit Entnahme von biologischen Proben ist eine Herausforderung. Durch enge Kooperation mit Geburtskliniken sowie mit niedergelassenen Ärztinnen und Ärzten kann die Teilnahme gesichert werden. Nabelschnurblutentnahme kann ein Hindernis für die Teilnahme darstellen, insbesondere wenn gleichzeitig die Geburtsklinik eine alternative Option für die Nabelschnurblutentnahme anbietet.

## Background

Prospective birth cohort studies, comprising follow up of children from pregnancy or birth over a long period of time and collecting various biological samples at different times through life-course, offer a promising approach for research on the etiology of various diseases [1]. There is growing evidence that intrauterine and early-life exposures may have an important influence on the development of various chronic diseases, and there is an increasing attention to the life-course approach in epidemiology [2], [3], [4]. According to the World Health Organization (WHO) environmental exposures are responsible for as much as 24% of avoidable global disease burden (healthy life years lost) [5]. A direct determination of exposures through the collection of biological samples is a key aspect and simultaneously a critical component in epidemiological studies on maternal and child health. This particularly applies for research regarding prenatal exposures to various environmental factors as well as for DNA extraction. Hence most of the existing birth cohort studies aim at collecting umbilical cord blood and other biological samples [1]. 

There have been long-ongoing population-based birth cohort activities as documented in the online inventory located at www.birthcohorts.net, providing an overview of 56 European pregnancy and birth cohorts fulfilling the inclusion criteria and representing 19 countries registered in a freely accessible database. The database is a useful tool for identification of birth cohort studies, with the majority of birth cohorts located in Northern and Western Europe [6]. A notable birth cohort activity in Europe is the so called Global Allergy and Asthma European Network (GA²LEN). GA²LEN was initiated to identify and compare European birth cohorts on asthma and atopic diseases, common chronic childhood diseases. Up to date, 18 European birth cohorts have been identified within GA²LEN and a common database containing their respective study characteristics has been established [7], [8].

Regarding the investigation of rare diseases, e.g. childhood leukemia (CL) broad collaborations, such as the International Childhood Cancer Cohort Consortium (I4C) open up the possibility to comprehensively investigate causes by pooling data from single prospective birth cohorts. Sufficiently large data sets will allow better understanding of the interplay between environmental and genetic factors [9]. 

Renewed research efforts on the etiology of CL focus on the multifactorial etiology of the disease. CL is the most common type of childhood cancer, accounting for 30% of all cancers diagnosed in children younger than 15 years in most Western populations [10], [11]. Despite extensive efforts to investigate and evaluate causes for CL, there are few consistently established etiologic factors, among them ionizing radiation [12], [13], [14], [15]. Various other possible risk factors are being discussed in relation to CL, i.e. birth weight and maternal or early childhood infections [12], [16], [17], [18], [19], [20], [21], [22], [23], [24], [25], [26], [27], [28], [29], [30], [31], [32], [33], [34]. However, prospective research approaches (i.e. birth cohort studies) with sufficiently large data sets are needed for further etiologic research, since consistent evidence for many of these factors and their contribution to the etiology of CL is still missing [9].

In Germany, several small to mid-size birth cohorts have been conducted or are currently under way, but a large scale and comprehensive birth cohort study has yet to be established. Some of the existing birth cohorts started enrollment at the time of birth or later, and not during pregnancy [35], [36], [37]. The focus of the majority of German birth cohorts is on allergies and atopic diseases [35], [36], [37]. A conceptual framework for a German national birth cohort for environmental health research was recently commissioned by the German Federal Environment Agency [38].

This one-year feasibility study for a birth cohort with prospective sampling of expectant women and their future newborns aimed to investigate the willingness of pregnant women to participate in a birth cohort study involving the donation of blood and umbilical cord blood samples for research purposes. The overall aim of the study was to develop practice-based research recommendations for a possible German birth cohort study that could be used to further study risk factors for leukemia. The study was funded by the German Federal Office for Radiation Protection (BfS).

## Methods

### Study design and setting

The study was conducted in Bremen (Germany) from January 2012 to March 2013. The recruitment of pregnant women was carried out in cooperation with one maternity hospital in the federal city-state of Bremen. Furthermore we contacted 22 gynecologists with an office in the vicinity of the cooperating hospital in order to enroll women in the study. The study region covers a geographical area of 419.38 km² with 654,774 residents and 5,657 deliveries in 2012. Based on 2012 data, approximately 1,995 of these deliveries were in the cooperating hospital and approximately 1,000 births within the recruitment period. 

This feasibility study included a review of scientific and technical foundations and the subsequent conduct of a study with pregnant women, the sampling and asservation of maternal blood and umbilical cord blood samples in an appropriate blood bank and the clarification of ethical and data protection requirements (obtaining written approval). Cooperation with a maternity hospital and a stem cell bank were established. Basic descriptive data on the study are provided below.

### Recruitment of participants

Pregnant women were eligible for recruitment if (i) their expected date of delivery was during the recruitment phase (between September 2012 and February 2013), (ii) they planned to give birth at the cooperating maternity hospital in Bremen and (iii) their knowledge of the German language was sufficient to understand study materials, details of participation and to fill out the self-administered questionnaire. Women with limited or no knowledge of the German language could not be enrolled in the study. 

Expectant women were recruited during the last trimester of pregnancy, either during one of their prenatal care visits at their primary care gynecologist or later in the hospital. If the women were accompanied by their partners, the couple was jointly informed and asked to participate in the study. Women/couples were initially informed about the feasibility study by primary care gynecologists, hospital staff (midwives, physicians) or by a study nurse. We provided study information and materials (i.e. study flyer, study information, consent form and a guideline for enrollment of participants) for addressing pregnant women/couples. Those who showed interest in the study were then asked to fill out a consent form stating that they agreed to be contacted by the research team. Thereafter, the women/couples had the opportunity to discuss the study in detail with a member of the project team, and to decide on participation. 

### Data collection

Data collection instruments included (i) a newborn documentation sheet filled out by the hospital staff, (ii) a self-administered questionnaire to be completed by the expectant mothers covering potential risk factors on childhood leukemia, general information about the pregnancy and the period prior to birth and (iii) a copy of the pregnancy record book (Mutterpass). The latter is a document issued to every woman in Germany following the initial ascertainment of a pregnancy. The pregnancy record book is used during routine prenatal care to document relevant information about the course of pregnancy (e.g. data about possible risk factors, physical examinations, prenatal ultrasound examinations) and on maternal and child health (e.g. immunization status for rubella and toxoplasmosis, estimated fetal birth weight). (For further details see Table 1 (Tab. 1)). In addition, we sent a short questionnaire on acceptance of the self-administered questionnaire, including few items on comprehensibility and duration of completion. We also asked what respondents liked and disliked about the questionnaire.

To allow for the assessment of non-response bias, expectant women not wishing to participate in the study were asked to complete a non-responder questionnaire covering few key demographic details, such as marital status, family size, school and professional education and family income. Furthermore, reasons for non-participation were asked.

### Use of incentives

To assess the influence of incentives on participation rates, we used a pragmatic approach to determine which group the pregnant women would be assigned to. Half of the cooperating primary care gynecologists offered the monetary incentive of 30 EUR while informing the pregnant women about the study, and the other half did not. In the hospital, potential participants were informed about the incentive every second week during the recruitment period. The other half of potential participants received the 30 EUR after their participation.

To compensate for their time the involved primary care gynecologists and hospital staff (physicians, midwives) received monetary incentives for each successfully recruited woman (20 EUR) and also for each non-responder with completed questionnaire (10 EUR). To further increase acceptability and encourage participation we established a telephone hotline for participants, primary care gynecologists and hospital staff. We kept close contact to families and to collaborators, e.g. via birthday and Christmas cards. 

### Collection and storage of biological samples

As the cooperating maternity hospital already had an established cooperation with a stem cell blood bank, we adopted the existing procedures used for the collection of maternal as well as umbilical cord blood samples for genetic and biochemical analyses. To ensure standardized biological sample collection, trained hospital staff collected the biological samples at the time of birth. The stem cell bank was responsible for the transportation, processing and storage of the biological samples and also provided the collection set for umbilical cord blood and maternal blood samples. All study-related samples were pseudonymised before storage. 

### Data management and data analyses

All data were directly transferred into the study data base, where immediate automated data completeness and plausibility checks were done by specialists in medical informatics and medical documentation. In addition a range of strategies were utilized to maximize the quality of data collection. This included validation checks for consistency and completeness of routinely collected data, training of members of the study team and training of clinical and project staff.

### Ethics approval and consent 

The study was approved by the Ethics committee of the local chamber of physicians in Bremen (Bremen Medical Association). All participants gave written consent prior to being included in the study. A hotline number for queries was included in the study information and letter. Written consent was provided for interview participation, umbilical cord blood and maternal blood samples, copying of pregnancy record books and if more detailed information on birth was needed, for consultation of hospital staff. Analyses were executed exclusively at group level. Individual results of this study were not available for participants. 

## Results

Of the 22 contacted primary care gynecologists with an office in the vicinity of the cooperating hospital, 8 enrolled pregnant women for the study, including one joint Doctor’s office with three doctors. 9 of those who did not participate gave lack of time as the reason. The remaining 5 primary care gynecologists did not give any reason for non-participation. A total of 200 eligible women were asked to participate in the study. About 130 women (the exact number cannot be determined due to visitations of delivery room, antenatal classes, etc. with varying numbers of participants) were addressed in hospital setting and 70 in gynecological practices. Overall 48 (24%) women agreed to participate in the study. 34 women were enrolled by primary care gynecologists and 14 within the hospital. The number of women enrolled via individual primary care gynecologists varied greatly and ranged from 1 to 38 invited participants. 26 of the 34 women were recruited by one primary care gynecologist alone, whereas the remaining 7 primary care gynecologists recruited 8 women in total. 12 of the 14 hospital enrollments were done by project members in the context of antenatal care (i.e. midwife consultations, visitation of delivery rooms or antenatal classes). 11 of the 12 enrollments done by project members were recruited during a personal conversation after midwife consultations or antenatal classes.

41 women consented to the collection of umbilical cord blood and maternal blood samples and samples could be stored for 22 of them. Due to complications or emergencies surrounding birth, biological samples could not be stored for 19 women. The prenatal self-administered questionnaire was available for all of the 48 participating women. Newborn documentation sheets were available for 46 children and pregnancy record books for 36 women. Table 2 (Tab. 2) gives an overview of the basic characteristics of the participating mothers and children.

The use of incentives did not influence participation rates of primary care gynecologists in this feasibility study. Monetary incentives offered to potential participants were also not associated with a higher response rate in our study. Approximately 100 women were offered 30 EUR while informed about the study and 17 agreed; whereas 31 out of the other 100 women agreed to participate without the offer of monetary incentive while informed about the study. 

40 participants filled out the short questionnaire on acceptance of the self-administered questionnaire. 35 women rated the comprehensibility as “good” or “very good”, 29 scored the duration of completion as “good” or “very good” and 33 women were “satisfied” or “very satisfied” with the questionnaire. 36 of all respondents stated that they are willing to complete a further comparable questionnaire. 19 participants filled out the question regarding the dislikes/improvement of the questionnaire; the most common answer, stated by 5 women, was that the completion time of the questionnaire was too long.

Only limited data on non-responders was obtained. 6 potential participants not wishing to participate filled out the non-responder questionnaire. Reasons given for non-participation were (i) uncertainty whether or not the full study would be conducted, (ii) the fact that no specific use of the samples collected in this feasibility study could be given, and (iii) the fact that the women/couples were not willing to decide for their children whether or not genetic information (cord blood) can be stored for research purposes. 

## Discussion and recommendations

### Main findings

For the planning of large birth cohort studies it is important to understand different recruitment processes in the specific social as well as in the health care context. This study aimed to provide background data and tested methods for the establishment of a future German birth cohort study. We conclude that two main factors related to the type of study we conducted negatively influenced participation of women/couples. Firstly, as this was a feasibility study, there was uncertainty as to whether or not a full study would be realized. Linked to this, women/couples did not want to donate umbilical cord blood samples in the context of a feasibility study, in which the exact purpose of the donated biological samples was not defined. Furthermore, the willingness to donate umbilical cord blood samples was influenced by three competing alternatives. During the course of the study, we found out that the hospital routinely offers private storage of cord blood samples for possible personal future use, as well as storage in a public stem cell blood bank. This presented competing alternatives to the research purpose offered in our study. Our request for donation for research purposes made up the third alternative. 

### Comparison with other studies and implications for future research

Nevertheless, about 24% of the invited women/couples were willing to participate in this feasibility study which we regard as low to moderate. Participation rates reported from other birth cohort studies were 75% within the SNiP study [37] and 45% within the Norwegian Mother and Child Cohort (MoBa) [39]. 85% of the eligible population were included in the Avon Longitudinal Study of Parents and Children (ALSPAC) [40]. Despite the competing interests regarding the cord blood collection, 41 women consented to the collection of umbilical cord blood and maternal blood samples for research purposes and samples could be stored for 53.7% of them. Our findings regarding biosample collection are somewhat lower, but generally in line with those from other German birth cohort studies. In the LISAplus study umbilical cord blood samples could be collected for 64.0% (n=1,983/3,097) of the initially recruited neonates, while for the GINIplus study umbilical cord blood samples could be collected for 24.1% (n=1,441/5,991) [36]. Availability of cord blood samples in the SNiP study was 81% (n=5,531/6,828) [37] and 84.1% in MAS-90 (n=6,398/7,609) [35]. 

The prenatal and early postnatal phases are the periods where most critical events occur, but also where so far the largest gaps in knowledge exist [1]. A particular challenge for birth cohorts is the early recruitment of pregnant women. To ensure detailed assessment of exposures during the prenatal phase and in terms of international comparability, recruitment within the first trimester is desirable [41]. Hence, birth cohorts such as the Danish National Birth Cohort (DNBC), the US National Children’s Study (NCS) and the MoBa study enroll women early during pregnancy [42], [43], [44]. The NCS initially planned a pre-conception household based recruitment. They found that response and yield of pregnancies was markedly lower than expected and changed their originally proposed recruitment strategy to a more traditional one, i.e. recruitment via prenatal care sites [44]. With the existing structures of the German health care system it is questionable whether very early recruitment is feasible. In addition, enrollment before pregnancy or during the first trimester might not be well accepted by potential participants, given the chance of early negative pregnancy outcomes, such as pregnancy loss [45]. Due to the time frame of our study, enrollment did not start earlier than the third trimester of pregnancy. Overall, there is little experience in Germany with cohort recruitment of pregnant women. Most available German birth cohort studies i.e. LISAplus and GINIplus [36], SNiP [37] and MAS [35] started enrollment only at the time of birth. Within the on-going LIFE Child study, the Life Child BIRTH study aims at recruiting up to 2,000 pregnant women at 24–26 weeks of gestation. The overall recruitment approach relies on community-based collaborative network of university hospitals, local clinics, public health centres etc. [46].

For our study, we identified two effective recruitment approaches. The first approach was through direct contact with expecting women/couples in the context of antenatal care (i.e. midwife consultations) by project members. Secondly, particularly motivated primary care gynecologists are a major asset for effective recruitment: in our study, the majority of participating women were enrolled by one of the participating primary care gynecologists. The reasons why this one primary care gynecologist was so much more effective in engaging pregnant women than compared to the others may be due to the fact that it was a practice for prenatal medicine and ultrasound diagnostic. Thus this one gynecologist may have seen more pregnant women as compared to the other participating gynecologists, due to special examinations offered, e.g. ultrasound examinations with 3D/4D imaging, and referrals. A strategy of prospectively identifying and motivating key recruiters (e.g. particularly interested primary care gynecologists or specialists) to ensure success of recruitment may be an option for the future. An advantage of recruitment through primary care gynecologists is that the women could be addressed early in pregnancy, as they will have their first antenatal care visit during the first trimester of pregnancy. In contrast, the women normally will attend the hospital at a later stage to register for delivery, i.e. within the third trimester of pregnancy. Further, personal contact between study team members and participants at enrollment played an important role. This is in line with results from other European birth cohorts [47], [39]. In this respect, the deployment of permanent study personnel in maternity units needs to be considered.

The establishment of recruitment and other study procedures, in particular within the hospital, took up quite a lot of time. Since the cooperating institutions (e.g. maternity unit, gynecologists) disseminate the first information about the study and are in a position to motivate pregnant women to participate in the study, it is essential to establish close collaboration with primary care gynecologists and maternity hospitals and to ensure that they are involved in the study at an early stage. Incentives seem to be decisive only to a limited extent for specific population groups, e.g. those with low socio-economic status (SES).

An important issue emerging in our study concerned the three alternatives concerning cord blood collection. The possibility of combining the three: (i) private storage for possible personal future use, involving major personal expenses, (ii) storage in a public stem cell blood bank with the advantage of availability for all patients and (iii) donation for research purposes, should be considered when planning similar studies. This then leads to the aspect of informed or broad consent. This is a major issue in terms of providing adequate and precise information to potential participants and ensuring appropriate consent procedures addressing the storage and analyses of biological samples. So far, ethical review boards in Germany generally require informed consent regarding a specified use of biosamples, so that the prospective participant gets a clear idea of what the donated samples will be used for. Newly planned analyses have to be presented and assessed separately by the ethical review board. At present there are no specific statutory provisions for biobanks in Germany, so that there is no clear legal basis for a potential large national birth cohort including cord blood sampling. Usually it is not possible to inform the donors in advance about the precise purposes of use and of the duration of storage. Due to the growing importance of biobanks for research purposes and to solve the unclear legal situation the German Ethics Council recommended a “five-pillar-concept”. Among other things it is proposed that the narrowly limited use of data and duration of storage of samples should be replaced by legislation exclusively for scientific research and the introduction of thorough biobank secrecy [48]. For a potential large-scale birth cohort, a study-specific biobanking concept will be essential, perhaps making use of ongoing developments in the framework of the German National Cohort [38], [49].

To enable confidence building in a large cohort the skillful use of public relations is essential. We used study specific flyers and posters and established a study hotline. Existing community networks may be very useful for public relations. A study website can be used both for information purposes and for conducting web-based follow up [50]. In the future web-based questionnaires and recruitment through the internet may become increasingly important. The NINFEA birth cohort from Italy demonstrated that recruitment via internet and web-based questionnaires, prenatal and six and 18 months after delivery, was well accepted by the users [51]. The Danish National Birth Cohort (DNBC) used email-based and web-based questionnaires within their 7-year follow-up [52]. Various other birth cohorts are planning to use web-based questionnaires, e.g. PRIDE-study (Netherlands).

### Potential limitations

A limitation of our one-year feasibility study was that we could only involve women whose knowledge of the German language was sufficient to understand study materials and details of participation. The recruitment process of a possible national birth cohort study should however involve migrant sensitive approaches and approaches to enroll hard-to-reach groups. Furthermore, in order to reach different populations/target groups and to increase overall response, a number of different survey instruments should be introduced, e.g. paper-and-pencil as well as web-based questionnaires. To include a broad range of potential participants, the recruitment should be in cooperation with multiple institutions, e.g. maternity units, primary care gynecologists, community services or other services for pregnant women.

## Conclusions

In general willingness of parents to be enrolled in this feasibility study for a birth cohort with biosampling was low to moderate. Pregnant women seemed to be more willing to participate in a birth cohort when approached by particular motivated key recruiter and when personal contact through project members was established, i.e. in a hospital setting by a study nurse, than compared to recruitment attempts in most (antenatal) primary care practices (with the exception of one practice). As shown in previous birth cohorts the vast majority of women who agreed to join the study also agreed to the cord blood collection, which is promising for the conduct of a possible national birth cohort study in Germany. However the request for umbilical cord blood collection may hinder participation, especially when maternity hospitals offer an alternative option for cord blood donation. Account should be taken in regard to competing alternatives concerning the cord blood collection and possibilities to combine these interests. The survey instruments and procedures established for the feasibility study were well received and materials can be made available to other researchers on request. It might be an option for the future to divide the relatively long questionnaire into several smaller parts to reduce completion time and burden of participation. For the purposes of implementing a possible national birth cohort study in Germany further thorough pretesting of recruitment is essential, e.g. future projects could examine further recruitment strategies in the social context, e.g. community-based recruitment. Furthermore it would be interesting to examine if incentives given directly (and only) to doctor’s receptionists or assistants in the primary care practice may improve participation of pregnant women during their first antenatal care visit.

## Notes

### Funding

The study was funded by the German Federal Office for Radiation Protection (funding code: 3611S70020).

### Competing interests

The authors declare that they have no competing interests.

## Figures and Tables

**Table 1:**
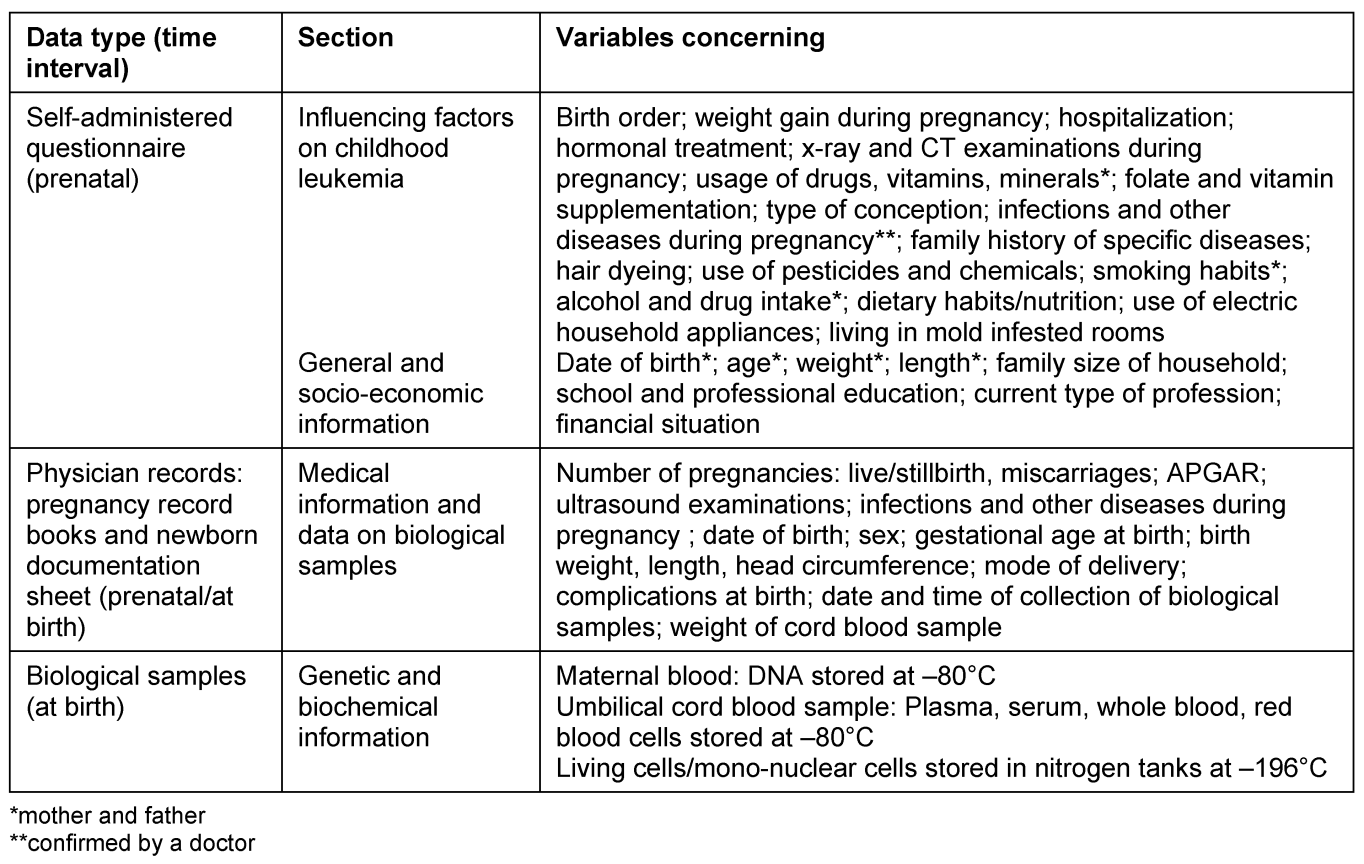
Data collection, variables and source of information

**Table 2:**
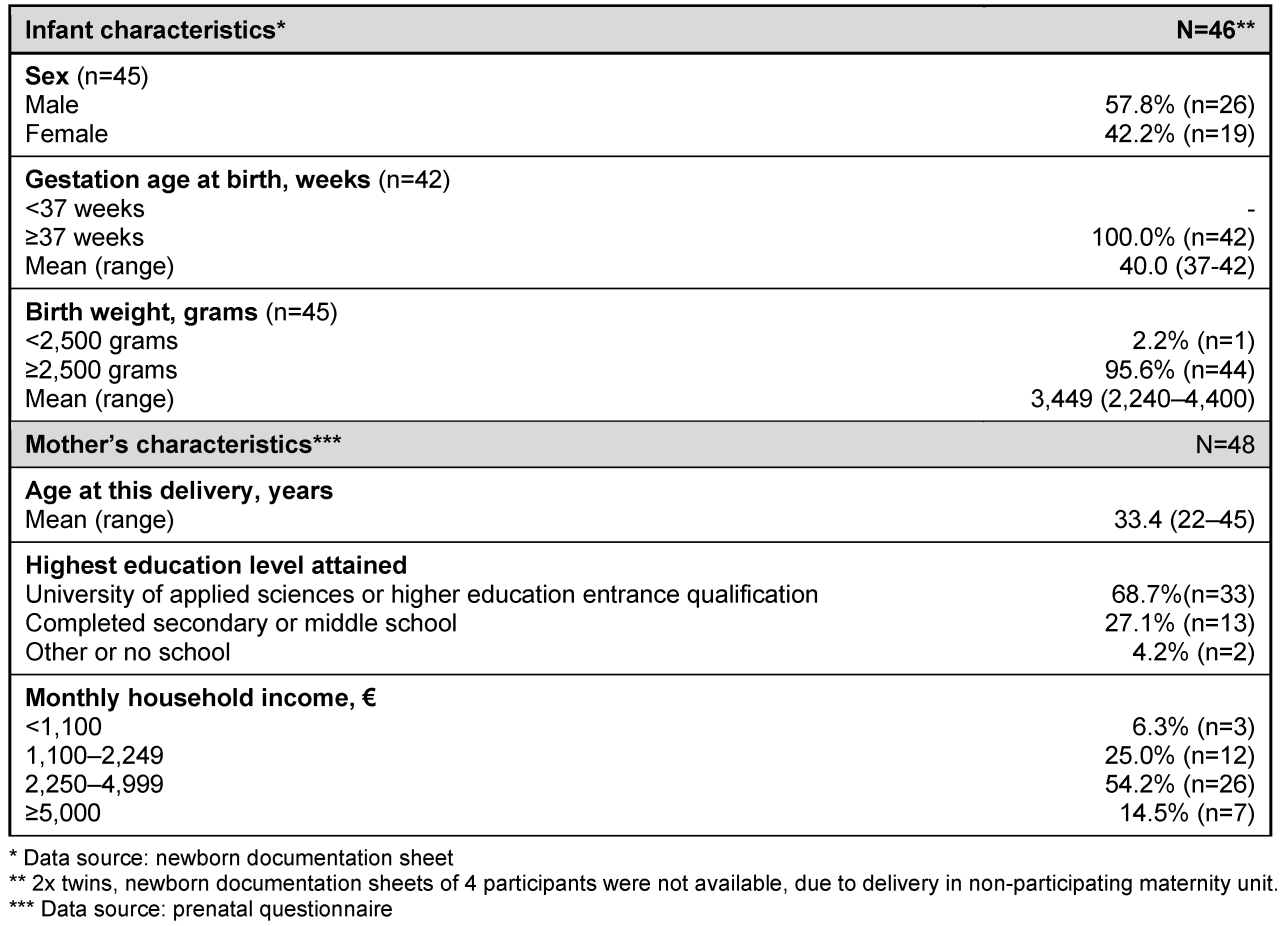
Baseline characteristics and demographic data on mothers and their newborns
